# 4-Methyl-*N*-[(*E*)-4-methyl-1-(4-methyl­phenyl­sulfon­yl)-1,2-dihydropyridin-2-yl­idene]benzene­sulfonamide

**DOI:** 10.1107/S1600536810025158

**Published:** 2010-07-03

**Authors:** Masoumeh Tabatabaee, Mitra Ghassemzadeh, Liela Hesami, Bernhard Neumüller

**Affiliations:** a Department of Chemistry, Islamic Azad University, Yazd Branch, Yazd, Iran; b Chemistry & Chemical Engineering Research Centre of Iran, Tehran, Iran; c Fachbereich Chemie, Universitat Marburg, Marburg, Germany

## Abstract

The reaction of 2-(amino­meth­yl)pyridine and 4-toluene­sulfonyl chloride in CH_2_Cl_2_ at pH 8 led to the title compound, C_20_H_20_N_2_O_4_S_2_. The aromatic rings are almost perpendicular to each other and the dihedral angles between the aromatic ring planes are 74.33 (9) (central pyridine *versus* benzene ring of the tosyl group bonded to the imine functionality), 73.77 (6) (pyridine *versus* benzene ring of the tosyl group bonded to pyridinic N atom) and 79.83 (9)° (benzene rings of tosyl groups). In the crystal structure, inter­molecular aromatic π–π stacking inter­actions [centroid–centroid separation = 3.6274 (14) Å] help to consolidate the packing.

## Related literature

For sulfonamide compounds, see: Maren (1967 ▶); Supuran *et al.* (1999 ▶); Culf *et al.* (1997 ▶); Kremer *et al.* (2006 ▶). For 2-amino-methyl­pyridine sulfonamide derivatives, see: Beloso *et al.* (2003 ▶, 2004 ▶, 2005 ▶, 2006 ▶). For related N and S-containing compounds, see: Tabatabaee *et al.* (2006 ▶, 2007 ▶, 2008 ▶, 2009 ▶).
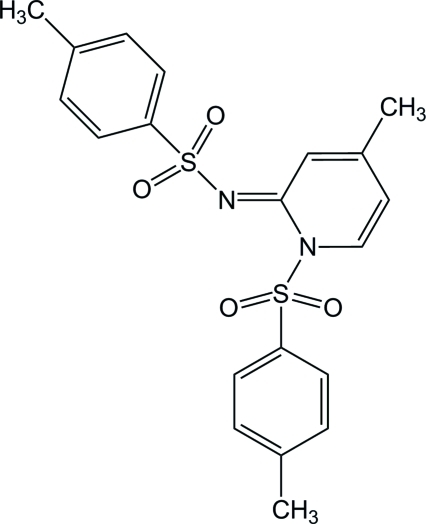


## Experimental

### 

#### Crystal data

C_20_H_20_N_2_O_4_S_2_
*M* *_r_* = 416.50
Orthorhombic, 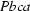

*a* = 14.888 (1) Å
*b* = 13.884 (1) Å
*c* = 18.843 (1) Å
*V* = 3894.9 (4) Å^3^
*Z* = 8
Mo *K*α radiation
μ = 0.30 mm^−1^
*T* = 100 K
0.21 × 0.12 × 0.08 mm

#### Data collection

Stoe IPDS-2 diffractometer
Absorption correction: integration (*X-RED32*; Stoe & Cie, 2002 ▶) *T*_min_ = 0.78, *T*_max_ = 1.0
39760 measured reflections
3785 independent reflections
2634 reflections with *I* > 2σ(*I*)
*R*_int_ = 0.139

#### Refinement

*R*[*F* ^2^ > 2σ(*F* ^2^)] = 0.044
*wR*(*F* ^2^) = 0.094
*S* = 0.94
3785 reflections
301 parameters
H atoms treated by a mixture of independent and constrained refinement
Δρ_max_ = 0.26 e Å^−3^
Δρ_min_ = −0.28 e Å^−3^

### 

Data collection: *X-AREA* (Stoe & Cie, 2002 ▶); cell refinement: *X-AREA*; data reduction: *X-RED32* (Stoe & Cie, 2002 ▶); program(s) used to solve structure: *SIR92* (Altomare *et al.*, 1993 ▶); program(s) used to refine structure: *SHELXL97* (Sheldrick, 2008 ▶); molecular graphics: *SHELXTL-Plus* (Sheldrick, 2008 ▶); software used to prepare material for publication: *WinGX* (Farrugia, 1999 ▶).

## Supplementary Material

Crystal structure: contains datablocks I, global. DOI: 10.1107/S1600536810025158/bh2287sup1.cif

Structure factors: contains datablocks I. DOI: 10.1107/S1600536810025158/bh2287Isup2.hkl

Additional supplementary materials:  crystallographic information; 3D view; checkCIF report

## Figures and Tables

**Fig. 1.**
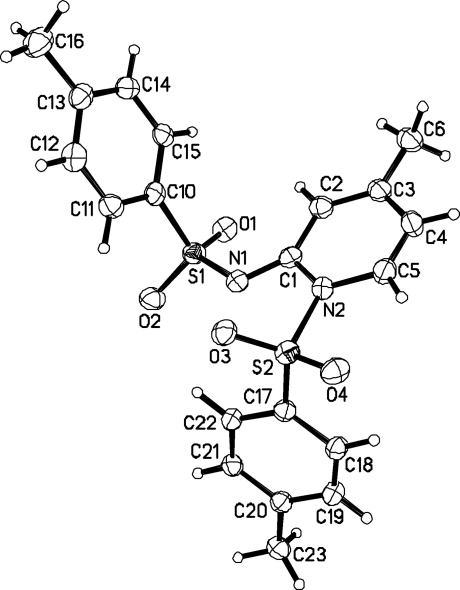
The general view of the title compound. Non-H atoms are represented with thermal ellipsoids (p = 50%).

**Fig. 2.**
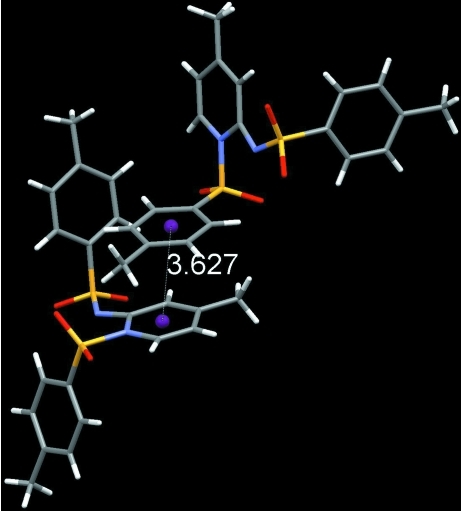
Representation of π–π stacking in the crystal.

## supplementary crystallographic information

### Comment

The sulfonamides represent an important class of biologically active compounds (Supuran *et al.*, 1999). There are several sulfonamide-based groups of drugs. Aromatic sulfonamides are strong inhibitors of carbonic anhydrase (Maren, 1967) and are of pharmacological value because of their effects on various physiological reactions ultimately involving bicarbonate. Some 10,000 structurally different sulfonamides have been synthesized as a result of the discovery of the antibacterial properties of sulfanilamide. The practice of synthesizing numerous structurally related compounds in an effort to find some that are more efficient or have fewer side effects than those already available is very important for the pharmaceutical industry. Moreover, sulfonamides containing different donor atoms find use in coordination chemistry. Recently, synthesis and crystal structure of several sulfonamide ligands with heterocyclic amines and their complexes have been reported (Culf *et al.*, 1997; Kremer *et al.*, 2006; Beloso *et al.*, 2003, 2004, 2005, 2006). In continuation of our recent work on synthesis of new ligands with S and N donor atoms (Tabatabaee *et al.*, 2006, 2007, 2008, 2009), in this paper we wish to report our results on the synthesis and crystal structure of a bisulfonamide compound, which resulted from 2-amino-4-methylpyridine and 4-toluenesulfonyl chloride. Reaction of one molecule of 4-toluenesulfonyl chloride with the NH_2_ group of 2-amino-4-methylpyridine led to corresponding sulfonamide. The resulting sulfonamide shows imido-amido tautomerism. Imido-amido tautomerism has been reported for some sulfonamide compounds (Beloso *et al.*, 2003, 2004, 2005). Reaction of another molecule of 4-toluenesulfonyl chloride with endocyclic NH group in imido form led to the title molecule.

In the molecule (Fig. 1), the bond lengths and angles are unexceptional. The molecule crystallizes in the orthorhombic system, space group *Pbca*. The basic six-membered ring skeletons are planar. *A*: C1/C2/C3/C4/C5/N2; *B*: C10···C15; *C*: C17···C22. The dihedral angles formed by the planes *A* and *B*, *A* and *C*, and *B* and *C*: are 74.33 (9), 73.77 (6), and 79.83 (9)%, respectively, indicating that aromatic rings are almost perpendicular to each other.

Figure 2 shows aromatic π–π stacking interactions between 6-membered rings with separation 3.6274 (14) for *Cg1*···*Cg3* (*Cg1*: ring A, *Cg3*: ring C).

### Experimental

2-amino-4-picoline (5 mmol) was added to a solution of 4-toluenesulfonyl chloride (10 mmol) in CH_2_Cl_2_ (30 ml). The pH of the resulting mixture was adjusted to 8 with an aqueous solution of sodium carbonate. The reaction mixture was stirred at room temperature. The progress of the reaction was monitored by thin layer chromatography (TLC), using ethyl acetate and *n*-hexane in 2:1 ratio as eluent. After completion of the reaction (12 h), water and ethyl acetate (50 ml 1:1) was added and the organic layer was separated. The solvent was evaporated and the solid residue was filtered, washed with cold CH_2_Cl_2_ (10 ml) and recrystallized from CH_2_Cl_2_.

### Refinement

All H atoms were detected in a difference map. Aromatic H atoms were refined freely, while methyl H atoms were idealized and refined as riding to their parent atom, with calculated isotropic displacement parameters.

### Crystal data

**Table:**

C_20_H_20_N_2_O_4_S_2_	*F*(000) = 1744
*M**_r_* = 416.50	*D*_x_ = 1.421 Mg m^−^^3^
Orthorhombic, *P**b**c**a*	Mo *K*α radiation, λ = 0.71073 Å
Hall symbol: -P 2ac 2ab	Cell parameters from 15000 reflections
*a* = 14.888 (1) Å	θ = 2.2–25.9°
*b* = 13.884 (1) Å	µ = 0.30 mm^−^^1^
*c* = 18.843 (1) Å	*T* = 100 K
*V* = 3894.9 (4) Å^3^	Blocks, colorless
*Z* = 8	0.21 × 0.12 × 0.08 mm

### Data collection

**Table:**

Stoe IPDS-2 diffractometer	3785 independent reflections
Radiation source: fine-focus sealed tube	2634 reflections with *I* > 2σ(*I*)
graphite	*R*_int_ = 0.139
ω scans	θ_max_ = 25.9°, θ_min_ = 2.2°
Absorption correction: integration (*X-RED32*; Stoe & Cie, 2002)	*h* = −18→16
*T*_min_ = 0.78, *T*_max_ = 1.0	*k* = −17→17
39760 measured reflections	*l* = −23→23

### Refinement

**Table:**

Refinement on *F*^2^	Primary atom site location: structure-invariant direct methods
Least-squares matrix: full	Secondary atom site location: difference Fourier map
*R*[*F*^2^ > 2σ(*F*^2^)] = 0.044	Hydrogen site location: inferred from neighbouring sites
*wR*(*F*^2^) = 0.094	H atoms treated by a mixture of independent and constrained refinement
*S* = 0.94	*w* = 1/[σ^2^(*F*_o_^2^) + (0.0461*P*)^2^] where *P* = (*F*_o_^2^ + 2*F*_c_^2^)/3
3785 reflections	(Δ/σ)_max_ = 0.006
301 parameters	Δρ_max_ = 0.26 e Å^−^^3^
0 restraints	Δρ_min_ = −0.28 e Å^−^^3^
0 constraints	

### Fractional atomic coordinates and isotropic or equivalent isotropic displacement parameters (Å^2^)

**Table:**

	*x*	*y*	*z*	*U*_iso_*/*U*_eq_	
N1	0.05540 (13)	0.20334 (16)	0.60238 (10)	0.0345 (5)	
C1	0.02054 (16)	0.17947 (17)	0.54034 (12)	0.0320 (5)	
C2	−0.05445 (18)	0.12038 (19)	0.52422 (13)	0.0350 (6)	
H21	−0.0872 (18)	0.094 (2)	0.5645 (14)	0.043 (8)*	
C3	−0.07778 (16)	0.09601 (18)	0.45642 (13)	0.0363 (6)	
C4	−0.02553 (18)	0.1336 (2)	0.40005 (14)	0.0404 (6)	
H41	−0.0383 (19)	0.118 (2)	0.3541 (14)	0.047 (8)*	
C5	0.04321 (18)	0.1926 (2)	0.41261 (13)	0.0380 (6)	
H51	0.0800 (18)	0.224 (2)	0.3798 (14)	0.043 (8)*	
N2	0.06665 (13)	0.21788 (15)	0.48155 (10)	0.0322 (5)	
C6	−0.15554 (18)	0.0317 (2)	0.44075 (15)	0.0458 (7)	
H61	−0.1993	0.0663	0.4115	0.074 (3)*	
H62	−0.1840	0.0119	0.4853	0.074 (3)*	
H63	−0.1344	−0.0255	0.4152	0.074 (3)*	
S1	0.01200 (4)	0.17417 (5)	0.67754 (3)	0.03399 (16)	
O1	−0.02748 (12)	0.07934 (12)	0.67874 (9)	0.0400 (4)	
O2	0.08054 (11)	0.19323 (14)	0.72943 (8)	0.0437 (5)	
C10	−0.07471 (16)	0.25859 (18)	0.69174 (11)	0.0311 (5)	
C11	−0.05235 (19)	0.3552 (2)	0.70311 (12)	0.0378 (6)	
H111	0.0090 (19)	0.375 (2)	0.7026 (13)	0.045 (8)*	
C12	−0.1196 (2)	0.4205 (2)	0.71944 (14)	0.0423 (6)	
H121	−0.1048 (18)	0.484 (2)	0.7293 (14)	0.045 (8)*	
C13	−0.20904 (18)	0.3922 (2)	0.72532 (12)	0.0388 (6)	
C14	−0.22968 (17)	0.2957 (2)	0.71211 (12)	0.0350 (6)	
H141	−0.2888 (16)	0.2719 (17)	0.7150 (11)	0.026 (6)*	
C15	−0.16385 (17)	0.22994 (19)	0.69476 (11)	0.0313 (5)	
H151	−0.1737 (16)	0.1651 (19)	0.6852 (12)	0.031 (6)*	
C16	−0.2808 (2)	0.4625 (2)	0.74675 (16)	0.0559 (8)	
H161	−0.3267	0.4656	0.7096	0.074 (3)*	
H162	−0.2541	0.5263	0.7534	0.074 (3)*	
H163	−0.3084	0.4412	0.7913	0.074 (3)*	
S2	0.15670 (4)	0.29710 (5)	0.49328 (3)	0.03332 (16)	
O3	0.12874 (12)	0.36943 (12)	0.54198 (9)	0.0383 (4)	
O4	0.17995 (12)	0.32336 (13)	0.42217 (8)	0.0397 (4)	
C17	0.24203 (16)	0.22493 (17)	0.52834 (11)	0.0300 (5)	
C18	0.29660 (16)	0.17449 (18)	0.48104 (12)	0.0333 (6)	
H181	0.2828 (16)	0.1738 (18)	0.4335 (13)	0.035 (7)*	
C19	0.36808 (17)	0.12187 (18)	0.50725 (13)	0.0345 (6)	
H191	0.405 (2)	0.088 (2)	0.4766 (14)	0.049 (8)*	
C20	0.38645 (17)	0.11905 (17)	0.57977 (12)	0.0316 (5)	
C21	0.33018 (16)	0.17011 (19)	0.62528 (12)	0.0336 (5)	
H211	0.3456 (18)	0.173 (2)	0.6730 (15)	0.046 (7)*	
C22	0.25832 (17)	0.22302 (19)	0.60086 (12)	0.0334 (6)	
H221	0.2183 (18)	0.259 (2)	0.6317 (14)	0.043 (7)*	
C23	0.46446 (17)	0.0628 (2)	0.60856 (13)	0.0384 (6)	
H231	0.5158	0.0691	0.5764	0.074 (3)*	
H232	0.4477	−0.0053	0.6126	0.074 (3)*	
H233	0.4807	0.0877	0.6555	0.074 (3)*	

### Atomic displacement parameters (Å^2^)

**Table:**

	*U*^11^	*U*^22^	*U*^33^	*U*^12^	*U*^13^	*U*^23^
N1	0.0301 (10)	0.0417 (12)	0.0318 (10)	−0.0014 (10)	0.0027 (8)	−0.0025 (9)
C1	0.0284 (12)	0.0321 (13)	0.0356 (12)	0.0052 (11)	0.0012 (10)	−0.0011 (10)
C2	0.0329 (13)	0.0338 (14)	0.0384 (13)	0.0031 (11)	0.0002 (11)	0.0002 (11)
C3	0.0323 (13)	0.0329 (14)	0.0437 (13)	0.0061 (11)	−0.0053 (11)	−0.0057 (11)
C4	0.0411 (15)	0.0447 (16)	0.0353 (14)	0.0059 (13)	−0.0082 (12)	−0.0085 (12)
C5	0.0384 (14)	0.0430 (16)	0.0326 (13)	0.0047 (13)	0.0020 (11)	−0.0025 (12)
N2	0.0314 (10)	0.0358 (12)	0.0294 (10)	−0.0007 (9)	0.0006 (8)	−0.0034 (8)
C6	0.0406 (15)	0.0410 (16)	0.0558 (16)	0.0016 (13)	−0.0085 (13)	−0.0077 (12)
S1	0.0302 (3)	0.0412 (4)	0.0305 (3)	0.0009 (3)	0.0005 (2)	0.0024 (3)
O1	0.0422 (10)	0.0346 (10)	0.0432 (9)	0.0007 (8)	0.0044 (8)	0.0043 (8)
O2	0.0350 (9)	0.0611 (13)	0.0352 (9)	0.0020 (9)	−0.0040 (7)	0.0040 (8)
C10	0.0358 (13)	0.0365 (14)	0.0208 (11)	−0.0008 (11)	0.0012 (9)	0.0022 (10)
C11	0.0359 (14)	0.0424 (16)	0.0351 (13)	−0.0063 (13)	0.0019 (11)	0.0016 (11)
C12	0.0512 (17)	0.0374 (16)	0.0385 (14)	−0.0017 (14)	0.0019 (12)	0.0003 (12)
C13	0.0440 (15)	0.0449 (17)	0.0274 (11)	0.0086 (13)	0.0011 (11)	0.0028 (11)
C14	0.0322 (13)	0.0482 (16)	0.0246 (11)	0.0000 (12)	0.0016 (9)	0.0058 (11)
C15	0.0329 (13)	0.0378 (15)	0.0232 (11)	−0.0031 (11)	−0.0014 (9)	0.0027 (10)
C16	0.0565 (19)	0.054 (2)	0.0571 (17)	0.0186 (16)	0.0006 (14)	−0.0007 (15)
S2	0.0315 (3)	0.0334 (3)	0.0350 (3)	0.0006 (3)	0.0029 (2)	0.0004 (3)
O3	0.0372 (10)	0.0310 (10)	0.0465 (10)	0.0024 (8)	0.0042 (8)	−0.0071 (8)
O4	0.0376 (10)	0.0441 (11)	0.0374 (9)	0.0026 (8)	0.0052 (7)	0.0088 (8)
C17	0.0292 (12)	0.0297 (13)	0.0311 (12)	−0.0023 (10)	0.0014 (9)	0.0005 (9)
C18	0.0338 (13)	0.0396 (15)	0.0264 (12)	0.0009 (11)	0.0032 (10)	0.0005 (10)
C19	0.0375 (14)	0.0353 (14)	0.0308 (12)	0.0042 (11)	0.0038 (11)	−0.0011 (11)
C20	0.0316 (13)	0.0293 (14)	0.0339 (12)	−0.0029 (11)	0.0008 (10)	0.0001 (10)
C21	0.0318 (13)	0.0415 (15)	0.0277 (11)	−0.0034 (11)	−0.0007 (10)	−0.0012 (10)
C22	0.0317 (13)	0.0377 (15)	0.0310 (12)	−0.0028 (11)	0.0046 (10)	−0.0065 (11)
C23	0.0371 (14)	0.0396 (15)	0.0384 (13)	−0.0002 (12)	−0.0057 (11)	−0.0002 (11)

### Geometric parameters (Å, °)

**Table:**

N1—C1	1.321 (3)	C13—C14	1.397 (4)
N1—S1	1.609 (2)	C13—C16	1.502 (4)
C1—N2	1.408 (3)	C14—C15	1.379 (4)
C1—C2	1.418 (4)	C14—H141	0.94 (2)
C2—C3	1.367 (3)	C15—H151	0.93 (3)
C2—H21	0.98 (3)	C16—H161	0.9800
C3—C4	1.416 (4)	C16—H162	0.9800
C3—C6	1.492 (4)	C16—H163	0.9800
C4—C5	1.333 (4)	S2—O3	1.4225 (17)
C4—H41	0.91 (3)	S2—O4	1.4310 (17)
C5—N2	1.390 (3)	S2—C17	1.748 (2)
C5—H51	0.93 (3)	C17—C22	1.388 (3)
N2—S2	1.748 (2)	C17—C18	1.395 (3)
C6—H61	0.9800	C18—C19	1.382 (3)
C6—H62	0.9800	C18—H181	0.92 (2)
C6—H63	0.9800	C19—C20	1.394 (3)
S1—O2	1.4378 (18)	C19—H191	0.93 (3)
S1—O1	1.4421 (19)	C20—C21	1.393 (3)
S1—C10	1.764 (3)	C20—C23	1.501 (3)
C10—C15	1.387 (3)	C21—C22	1.377 (4)
C10—C11	1.398 (4)	C21—H211	0.93 (3)
C11—C12	1.385 (4)	C22—H221	0.97 (3)
C11—H111	0.96 (3)	C23—H231	0.9800
C12—C13	1.392 (4)	C23—H232	0.9800
C12—H121	0.93 (3)	C23—H233	0.9800
			
C1—N1—S1	123.92 (18)	C15—C14—C13	121.4 (2)
N1—C1—N2	114.2 (2)	C15—C14—H141	116.4 (15)
N1—C1—C2	130.1 (2)	C13—C14—H141	122.2 (14)
N2—C1—C2	115.7 (2)	C14—C15—C10	120.0 (2)
C3—C2—C1	122.9 (2)	C14—C15—H151	125.1 (16)
C3—C2—H21	120.4 (16)	C10—C15—H151	114.9 (15)
C1—C2—H21	116.6 (15)	C13—C16—H161	109.5
C2—C3—C4	118.1 (2)	C13—C16—H162	109.5
C2—C3—C6	122.0 (2)	H161—C16—H162	109.5
C4—C3—C6	119.9 (2)	C13—C16—H163	109.5
C5—C4—C3	121.0 (2)	H161—C16—H163	109.5
C5—C4—H41	118.5 (18)	H162—C16—H163	109.5
C3—C4—H41	120.5 (18)	O3—S2—O4	119.67 (11)
C4—C5—N2	120.9 (2)	O3—S2—C17	111.94 (11)
C4—C5—H51	128.4 (16)	O4—S2—C17	108.91 (11)
N2—C5—H51	110.7 (17)	O3—S2—N2	107.53 (10)
C5—N2—C1	121.2 (2)	O4—S2—N2	103.14 (10)
C5—N2—S2	117.98 (17)	C17—S2—N2	104.15 (11)
C1—N2—S2	120.84 (15)	C22—C17—C18	121.2 (2)
C3—C6—H61	109.5	C22—C17—S2	120.67 (18)
C3—C6—H62	109.5	C18—C17—S2	118.02 (17)
H61—C6—H62	109.5	C19—C18—C17	119.1 (2)
C3—C6—H63	109.5	C19—C18—H181	121.0 (16)
H61—C6—H63	109.5	C17—C18—H181	119.8 (16)
H62—C6—H63	109.5	C18—C19—C20	121.1 (2)
O2—S1—O1	116.53 (11)	C18—C19—H191	120.5 (17)
O2—S1—N1	105.50 (11)	C20—C19—H191	118.4 (17)
O1—S1—N1	114.05 (11)	C21—C20—C19	118.1 (2)
O2—S1—C10	107.09 (11)	C21—C20—C23	120.5 (2)
O1—S1—C10	107.82 (11)	C19—C20—C23	121.4 (2)
N1—S1—C10	105.09 (11)	C22—C21—C20	122.2 (2)
C15—C10—C11	119.8 (2)	C22—C21—H211	119.6 (17)
C15—C10—S1	121.1 (2)	C20—C21—H211	117.9 (17)
C11—C10—S1	119.09 (19)	C21—C22—C17	118.4 (2)
C12—C11—C10	119.3 (3)	C21—C22—H221	123.4 (15)
C12—C11—H111	120.0 (17)	C17—C22—H221	118.3 (15)
C10—C11—H111	120.5 (17)	C20—C23—H231	109.5
C11—C12—C13	121.7 (3)	C20—C23—H232	109.5
C11—C12—H121	119.6 (17)	H231—C23—H232	109.5
C13—C12—H121	118.7 (17)	C20—C23—H233	109.5
C12—C13—C14	117.8 (2)	H231—C23—H233	109.5
C12—C13—C16	121.2 (3)	H232—C23—H233	109.5
C14—C13—C16	121.0 (3)		
